# The Application of the Cupping-Glass Recommended to Expedite the Discharge of Matter from Abscesses of the Scrofulous Kind

**Published:** 1819-12

**Authors:** Francis Bush

**Affiliations:** Member of the Royal College of Surgeons.


FOR THE LONDON MEDICAL AND PHYSICAL JOURNAL.

*The Application of the Cupping-glass recommended to expedite the Discharge of Matter from Abscesses of the scrofulous kind.* By FRANCIS BUSH, Esq. Member of the Royal College of Surgeons.

ABSCESSES of the subacute or scrofulous kind are generally indolent and tedious in their progress, but little under the control of constitutional means or the usual topical applications; and it often happens that the patient suffers a very protracted illness before the discharge of the matter takes place; and, where it is deep-seated, it frequently runs along the course of the contiguous muscles, producing extensive lesion and constitutional mischief. In the management of this disease I have been induced to adopt a plan to solicit the early discharge of the matter, which has been attended with a success beyond my most sanguine expectations. As I have nothing new to offer on inflammation, I shall not remark on the early treatment of abscess; but shall take up the subject where it is quite clear that matter is formed, and requires to be discharged.

One very striking advantage to be derived from the application of the measure I suggest, is, that you effect the discharge at the most depending part of the abscess. I shall subjoin two cases to illustrate my plan, which I am confident is capable of doing much service, if judiciously applied.

CASE I.—Mr. D. aged 24 years, was, after violent exercise, in the heat of July 1818, attacked with shivering, succeeded by deep-seated pain in the lumbar region. Leeches, general bleeding, and the usual methods of subduing local inflammation, were tried, without success. Matter formed; and it was invited to the surface by poultices of various kinds for some weeks, to but little purpose. In this state of the case, I thought, by taking off the atmospheric pressure from the most depending part of the tumor, I might produce some excitement in the parts, and effect an early discharge: for that purpose I applied a very small cupping-glass, and allowed it to remain on for an hour; it produced a little pain only. It was repeated in the evening; and for four successive days it was continued in a similar way. The removal of the glass was succeeded by a warm poultice. By these means, in five days, I effected a discharge from the most convenient part of the abscess, which might not have been produced, under the ordinary means of treatment, in as many weeks.

CASE II.—A young friend of mine was attacked with inflammation in the groin, which went on to suppuration: it was not attended with pain, and, after several weeks, there was no prospect of an opening being effected by the powers of nature. He was adverse to its being opened by the lancet; and, indeed, the indurated state of the parts rendered it improper in this stage of the case: a small cupping-glass was therefore applied daily for three or four days, and the parts covered with a poultice after its removal. The discharge of the matter was thus effected; and the parts healed in a very short time.

I considered both these cases as scrofulous. In phlegmonous abscesses the rapid progress of the disease renders this plan unnecessary, and the high irritability of the parts would make its application improper.

*Frome; October 1st,* 1819.

